# Disrupted functional brain network organization in patients with obstructive sleep apnea

**DOI:** 10.1002/brb3.441

**Published:** 2016-02-01

**Authors:** Bumhee Park, Jose A. Palomares, Mary A. Woo, Daniel W. Kang, Paul M. Macey, Frisca L. Yan‐Go, Ronald M. Harper, Rajesh Kumar

**Affiliations:** ^1^ Department of AnesthesiologyUniversity of California at Los AngelesLos AngelesCA90095; ^2^ UCLA School of NursingUniversity of California at Los AngelesLos AngelesCA90095; ^3^ Department of MedicineUniversity of California at Los AngelesLos AngelesCalifornia90095; ^4^ The Brain Research InstituteUniversity of California at Los AngelesLos AngelesCalifornia90095; ^5^ Department of NeurologyUniversity of California at Los AngelesLos AngelesCalifornia90095; ^6^ Department of NeurobiologyUniversity of California at Los AngelesLos AngelesCalifornia90095; ^7^ Department of Radiological SciencesUniversity of California at Los AngelesLos AngelesCalifornia90095; ^8^ Department of BioengineeringUniversity of California at Los AngelesLos AngelesCalifornia90095

**Keywords:** Functional magnetic resonance imaging, functional network, graph theory, spontaneous activity

## Abstract

**Introduction:**

Obstructive sleep apnea (OSA) subjects show impaired autonomic, affective, executive, sensorimotor, and cognitive functions. Brain injury in OSA subjects appears in multiple sites regulating these functions, but the integrity of functional networks within the regulatory sites remains unclear. Our aim was to examine the functional interactions and the complex network organization of these interactions across the whole brain in OSA, using regional functional connectivity (FC) and brain network topological properties.

**Methods:**

We collected resting‐state functional magnetic resonance imaging (MRI) data, using a 3.0‐Tesla MRI scanner, from 69 newly diagnosed, treatment‐naïve, moderate‐to‐severe OSA (age, 48.3 ± 9.2 years; body mass index, 31 ± 6.2 kg/m^2^; apnea–hypopnea index (AHI), 35.6 ± 23.3 events/h) and 82 control subjects (47.6 ± 9.1 years; body mass index, 25.1 ± 3.5 kg/m^2^). Data were analyzed to examine FC in OSA over controls as interregional correlations and brain network topological properties.

**Results:**

Obstructive sleep apnea subjects showed significantly altered FC in the cerebellar, frontal, parietal, temporal, occipital, limbic, and basal ganglia regions (FDR,* P* < 0.05). Entire functional brain networks in OSA subjects showed significantly less efficient integration, and their regional topological properties of functional integration and specialization characteristics also showed declined trends in areas showing altered FC, an outcome which would interfere with brain network organization (*P* < 0.05; 10,000 permutations). Brain sites with abnormal topological properties in OSA showed significant relationships with AHI scores.

**Conclusions:**

Our findings suggest that the dysfunction extends to resting conditions, and the altered FC and impaired network organization may underlie the impaired responses in autonomic, cognitive, and sensorimotor functions. The outcomes likely result from the prominent structural changes in both axons and nuclear structures, which occur in the condition.

## Introduction

Obstructive sleep apnea (OSA) is a common condition, characterized by recurrent episodes of partial or complete obstruction of the upper airway, with continued diaphragmatic efforts during sleep (Peppard et al. 2000). The disorder is accompanied by brain tissue injury, expressed as altered white matter integrity, free water content, brain metabolites, and regional gray matter volume in multiple brain regions, including the cerebellum, cingulate cortex, insular cortex, hippocampus, basal ganglia, thalamus, frontal regions, and pre‐ and post‐central sensorimotor sites, responsible for autonomic, cognitive, affective, and sensorimotor controls (Macey et al. 2002, 2008; Morrell et al. 2003, 2010; Torelli et al. 2011; Kumar et al. 2012, 2014b). The structural changes are accompanied by impaired functional magnetic resonance imaging (fMRI) responses to evoked autonomic, sensorimotor, and ventilatory challenges (Harper et al. 2003; Henderson et al. 2003; Macey et al. 2014), and changes in neurons, glia, and axons may alter overall resting‐state functional organization as well. Obstructive sleep apnea subjects show altered resting‐state functional connectivity (FC) based on voxel‐level independent component analysis (Zhang et al. 2013), and compromised resting‐state regional activity using regional homogeneity (Santarnecchi et al. 2013; Peng et al. 2014). Although previous resting‐state fMRI OSA studies address local brain dysfunction, they do not directly yield important macroscopic or topological changes, for example, how whole‐brain structures interact during resting states (i.e., connection weights among regions), and the complex network organization of these interactions (i.e., brain network shape).

Resting‐state fMRI (rs‐fMRI) procedures have been used to assess inter‐regional FC, a term which refers to temporal correlations between neuronal activity of anatomically distinct brain regions (Friston et al. 1994). The procedure identifies synchronized spontaneous low‐frequency (<0.1 Hz) fluctuation of blood oxygen level‐dependent (BOLD) signals across the brain in the resting state (Biswal et al. 1995; Lowe et al. 1998; Cordes et al. 2000). Based on consistent patterns across healthy subjects (Beckmann and Smith 2005; Damoiseaux et al. 2006; De Luca et al. 2006; Fox and Raichle 2007), resting‐state FC procedures have been applied widely in various functional brain network studies, ranging from psychiatric disorders to neurological conditions (Fox and Greicius 2010), as well as in exploration of human brain functions (Smith et al. 2009; Laird et al. 2013; Sadaghiani and Kleinschmidt 2013). The procedures may be useful in assessing functional brain networks in OSA subjects, since the condition is accompanied by severe behavioral and physiological sequelae.

In rs‐fMRI procedures, “graph‐theoretical” approaches have been used to characterize the complex system of functional brain networks, and to elucidate the topological properties of such networks (Bullmore and Sporns 2009, 2012). A brain network can be represented graphically, called a “brain graph,” which consists of a set of nodes (brain regions) and edges (connectivity between nodes) (Sporns et al. 2000; Sporns and Zwi 2004; Bullmore and Sporns 2009). Graph theory measures suggest that human brain networks are organized into modular systems, which are characterized by efficient integration of segregated brain regions through short paths, with low wiring costs consisting of a few densely connected hub regions in the whole brain (Watts and Strogatz 1998; Bullmore and Sporns 2009, 2012). Brain networks with high global efficiency contribute to cognitive processing through efficient integration among whole‐brain regions (Sporns and Zwi 2004), where some hubs (e.g., brain regions concentrated by large number of connections with the rest of whole brain) play a pivotal role showing high cost and are especially vulnerable to aberrant disease conditions (Crossley et al. 2014). These topological human brain attributes have been found both in anatomical networks using diffusion tensor imaging or cortical thickness assessments (Hagmann et al. 2007; He et al. 2007; Bassett et al. 2008; Iturria‐Medina et al. 2008; Gong et al. 2009), and in functional networks using MEG, EEG, or fMRI (Stam 2004; Eguiluz et al. 2005; Salvador et al. 2005; Achard et al. 2006; Stam and Reijneveld 2007).

In this study, our aim was to investigate the functional interactions and complex organizations across the whole brain in newly diagnosed, treatment‐naïve OSA subjects, relative to age‐ and gender‐comparable control subjects, using FC and graph‐theoretical measures. We hypothesized that OSA subjects would show intrinsically abnormal whole‐brain FC and deficient global integration and local segregation in functional brain organization within regions that control autonomic, affective, executive, sensorimotor, and cognitive functions over control subjects.

## Methods

### Design

We used a comparative cross‐sectional study design to assess resting functional interactions and the complex network organization of these interactions across the whole brain in recently diagnosed, treatment‐naïve OSA over control subjects.

### Subjects

We investigated 69 newly diagnosed, treatment‐naïve OSA and 82 age‐ and gender‐comparable control subjects. All OSA subjects had a moderate‐to‐severe diagnosis [apnea–hypopnea index (AHI) ≥15 events/h], and were recruited from the Sleep Disorders Laboratory at the University of California at Los Angeles (UCLA) Medical Center. Obstructive sleep apnea subjects were not taking any cardiovascular‐altering medications (e.g., *β*‐blockers, *α*‐agonists, angiotensin‐converting enzyme inhibitors, or vasodilators) or any mood‐changing drugs (e.g., selective serotonin reuptake inhibitors, hemodynamic‐altering, or metabolic‐altering drugs). No OSA subject had a history of neurological illness (e.g., stroke, heart failure) or psychiatric disorders other than the OSA condition. Control subjects were healthy, without any history of neurological issues, and were recruited from the UCLA campus and West Los Angeles area. We interviewed control subjects, as well as their sleep partners (~25%), when available, to determine the potential for sleep disordered breathing, and control subjects suspected of having such disturbed patterns, based on symptoms of snoring and gasping or with abnormal Pittsburgh Sleep Quality Index (PSQI) and Epworth Sleepiness Scale (ESS) scores, underwent an overnight PSG study (*n* = 3). We used OSA diagnosis criteria to categorize control subjects, who went for overnight PSG, either control or OSA (The Report of an American Academy of Sleep Medicine Task Force. 1999). Of three control subjects, one became OSA (AHI, 18 events/h) and two were in the same control pool (AHI < 3 events/h). Both OSA and control subjects were without any metallic implants, and had no conditions contraindicated for an MRI scanner environment. All participants gave written informed consent before data acquisition and study protocol was approved by the Institutional Review Board at the UCLA.

### Examination of sleep, mood, and anxiety symptoms

Sleep quality and daytime sleepiness were evaluated in OSA and control subjects using the PSQI and ESS questionnaires, respectively. The Beck Depression Inventory II (BDI‐II) was used to assess depressive symptoms, and the Beck Anxiety Inventory (BAI) was used to examine anxiety symptoms in OSA and control subjects. Both BDI‐II and BAI are self‐administered questionnaires (21 questions; each score ranges from 0 to 3), with total scores ranging from 0 to 63, based on mood or anxiety symptom severity.

### Magnetic resonance imaging

Brain imaging of all participants was performed using a 3.0‐Tesla MRI scanner (Siemens, Magnetom Tim‐Trio, Erlangen, Germany), with an 8‐channel phased‐array head coil. Foam pads were used on either side of the head to reduce head motion‐related artifacts during scanning. Rs‐fMRI data were acquired with an echo planar imaging (EPI)‐based blood oxygen level‐dependent (BOLD) sequence in the axial plane [repetition time (TR) = 2000 msec; echo time (TE) = 30 msec; flip angle (FA) = 90°; field‐of‐view (FOV) = 230 × 230 mm^2^; matrix size = 64 × 64; voxel size = 3.59 × 3.59 × 4.5 mm^3^; volumes = 59]. Rs‐fMRI data were acquired while participants laid resting with eyes open, without focusing any specific thoughts, and without sleeping for about 2 min (subjects were merely instructed not to sleep). High‐resolution T1‐weighted images were collected from each subject using a magnetization‐prepared rapid acquisition gradient echo (MPRAGE) pulse sequence (TR = 2200 msec; TE = 2.2, 2.34 msec; FA = 9°; FOV = 230 × 230 mm^2^; matrix size = 256 × 256, 320 × 320; voxel size = 0.9 × 0.9 × 1.0 mm^3^, 0.72 × 0.72 × 0.9 mm^3^). Proton density (PD) and T2‐weighted images were acquired in the axial plane, using a dual‐echo turbo spin‐echo pulse sequence (TR = 10,000 msec; TE1, 2 = 17, 134 msec; FA = 130°; matrix size = 256 × 256; FOV = 230 × 230 mm^2^; voxel size = 0.9 × 0.9 × 4.0 mm^3^). All MRI data were acquired at an intermediate time from 8:00 am to 2:00 pm.

### Data preprocessing

We used the statistical parametric mapping package (SPM8, Wellcome Department of Cognitive Neurology, London, UK) (Friston et al. 1994) and MRIcroN software for evaluation of images and for preprocessing of rs‐fMRI data. High‐resolution T1‐weighted, PD‐, and T2‐weighted images of all subjects were examined for any serious brain pathology, such as tumors, cysts, or major infarcts. Rs‐fMRI data were also assessed for imaging or head motion‐related artifacts before data processing. Obstructive sleep apnea and control subjects included in this study did not show any serious visible brain pathology, head motion‐related, or other imaging artifacts, which were checked through T1‐weighted, T2‐weighted, and PD‐weighted images.

Rs‐fMRI data preprocessing steps included realignment of EPI brain volumes for removal of any potential head motion, co‐registration to T1‐weighted images, and spatial normalization to a standard common space template using nonlinear transformation procedures. For rs‐fMRI analysis, we discarded the initial three brain volumes to avoid signal saturation issues, and used the remaining 56 EPI scans for analysis. No spatial smoothing was performed on the resting‐state fMRI data to avoid inflation of local connectivity and clustering (van den Heuvel et al. 2008).

### Functional network construction and analysis

Individual whole‐brain FC was determined from regional mean fMRI time series, extracted from 116 distinct regions, as defined by automated anatomical labeling (Tzourio‐Mazoyer et al. 2002), which consists of 90 cerebral brain regions (45 sites in each hemisphere) and 26 cerebellar areas (nine lobule regions in each hemisphere and eight vermis regions), as described in Table 1. For each regional mean fMRI time series, we applied the canonical signal processing procedures for calculating the resting‐state FC, as outlined previously (Weissenbacher et al. 2009). After removing effects of six rigid motions, their first derivatives, and global signal changes in white matter, cerebrospinal fluid, and whole brain from each time series, data were band‐pass filtered (0.009~0.08 Hz) using the fast Fourier transform (FFT) filter. Head motion effects are often an issue in any resting‐state FC study (Power et al. 2012; Van Dijk et al. 2012; Yan et al. 2013), and we added the first derivatives of the motion parameters as covariates to minimize signal changes from such motion (Power et al. 2012). Since removal of global signal still remains a controversial issue (Murphy et al. 2009; Chai et al. 2012), we performed additional FC analysis without global signal regression. Finally, we defined FC (edge) as an interregional correlation map among 116 preprocessed regional time series. To improve normality, we converted individual correlation maps into *z*‐scored maps with Fisher's *r*‐to‐*z* transformation. We compared the *z*‐scored maps edge‐by‐edge between OSA and control subjects using analysis of covariance (ANCOVA), with age and gender included as covariates to regress out age‐ and gender‐related differences. All resting‐state FC analyses were performed using MATLAB‐based custom software.

**Table 1:** Regional abbreviation table corresponding to 116 brain regions

Regions	Abbreviation	Regions	Abbreviation
Precental gyrus	PrCG	Supramarginal gyrus	SMG
Superior frontal gyrus (dorsolateral part)	SFGdor	Angular gyrus	ANG
Orbitofrontal gyrus (superior part)	OFGsup	Precuneus	PRCU
Middle frontal gyrus	MFG	Paracentral lobule	PCL
Orbitofrontal gyrus (middle part)	OFGmid	Caudate	CAU
Inferior frontal gyrus (opercular part)	IFGop	Putamen	PUT
Inferior frontal gyrus (triangular part)	IFGtr	Pallidum	PAL
Orbitofrontal gyrus (inferior part)	OFGinf	Thalamus	THL
Rolandic operculum	ROL	Heschl gyrus	HES
Supplementary motor area	SMA	Superior temporal gyrus	STG
Olfactory cortex	OLF	Temporal pole (superior part)	TPsup
Superior frontal gyrus (medial part)	SFGmed	Middle temporal gyrus	MTG
Orbitofrontal gyrus (medial part)	OFGmed	Temporal pole (middle part)	TPmid
Rectus	REC	Inferior temporal gyrus	ITG
Insula	INS	Cerebellar crus I	CRcr‐I
Anterior cingulate cortex	ACC	Cerebellar crus II	CRcr‐II
Middle cingulate cortex	MCC	Cerebellum III	CR‐III
Posterior cingulate cortex	PCC	Cerebellum IV‐V	CR‐IV
Hippocampus	HP	Cerebellum VI	CR‐VI
Para‐hippocampal gyrus	PHG	Cerebellum VIIb	CR‐VIIb
Amygdala	AMYG	Cerebellum VIII	CR‐VIII
Calcarine	CAL	Cerebellum IX	CR‐IX
Cuneus	CUN	Cerebellum X	CR‐X
Lingual gyrus	LING	Vermis I‐II	VM‐I
Superior occipital gyrus	SOG	Vermis III	VM‐III
Middle occipital gyrus	MOG	Vermis IV‐V	VM‐IV
Inferior occipital gyrus	IOG	Vermis VI	VM‐VI
Fusiform gyrus	FFG	Vermis VII	VM‐VII
Postcentral gyrus	PoCG	Vermis VIII	VM‐VIII
Superior parietal gyrus	SPG	Vermis IX	VM‐IX
Inferior parietal lobule	IPL	Vermis X	VM‐X

### Network analysis

Topological characteristics of functional brain networks of OSA and control subjects were investigated with graph‐theoretical analyses (Rubinov and Sporns 2010; http://www.brain-connectivity-toolbox.net/). Brain networks can be regarded as a graph, G = (N, E), which consists of a set of nodes N (brain regions), and a set of connections E (FC) (Bullmore and Sporns 2009). Individual brain networks were constructed with a threshold of FDR < 0.05; we retained edge weights, and referred those weights as being connected, if the values were significant, and otherwise set the values to zero and considered as being not connected. We also investigated each brain connection for network centrality (degree, strength, and betweenness), network segregation (clustering coefficient and local efficiency), and network integration (nodal and global efficiency) properties (Bullmore and Sporns 2009, 2012; Rubinov and Sporns 2010).

Degree for a brain region is defined as number of edges linking the node to rest of the network (Rubinov and Sporns 2010), and a higher degree value indicates functional hub role of that area to integrate with other brain regions. Strength for a brain site is defined as the sum of edge strengths linking the node to rest of the network (Rubinov and Sporns 2010), and larger the value shows greater use of connection strength in communication. Betweenness for a brain area is defined as the fraction of shortest paths between two sites in the network passing through the area (Rubinov and Sporns 2010), and higher value indicates that many numbers of the shortest path lengths pass through the region showing high influence in the network communication. Level of functional communication efficiency between two brain sites can be expressed as inverse of the shortest weighted path length, which is the weight sum in edges that must be traversed to go from one site to another (Latora and Marchiori 2001). Weighted efficiency for a region is defined as the mean of inverse weighed shortest path length to the rest of the network, and global efficiency is defined as the average nodal efficiency (Latora and Marchiori 2001; Achard and Bullmore 2007). Higher nodal efficiency or shorter path length may imply that a brain region communicates more efficiently with the rest of the brain. The level at which a network is organized into densely segregated nodes can be quantified using the clustering coefficient (Watts and Strogatz 1998). Weighted clustering coefficient for a node quantifies the number of existing edges among the node's neighbors (i.e., nodes linking the node) divided by all their possible edges, and higher values of a brain region highlight densely connected local structure among the neighboring areas. Efficiency related to the weighted clustering coefficient can be quantified as local efficiency by considering the weighted shortest path length within the neighbors (Rubinov and Sporns 2010). We additionally performed correlation analyses between graph‐theoretical measures and AHI values, indices as OSA disease severity.

### Statistical significance for group‐level comparison

To compare each edge weight between groups, the false discovery rate (FDR) was used to control for multiple comparisons across all edges at the *q*‐level of 0.05 (Genovese et al. 2002). Nodal graph‐theoretical measures between groups first were assessed with a threshold of FDR < 0.05 to address multiple comparison problem, and we did not find any significance. We then examined group‐level comparisons of all graph‐theoretical measures using the random permutation test in a nonparametric fashion (Nichols and Holmes 2002), for obtaining more accurate trends with weak level of significance, since altered regional FC can obviously affect topological properties of OSA subjects.

For each graph‐theoretical measure, we created a null distribution of t‐statistics from ANCOVA (covariates; age and gender), using 10,000 times randomly shuffled group labels, with the assumption of no significant differences between OSA and control group. We compared original *t*‐statistic values from ANCOVA with the null distribution, and considered the resulting values significant if they exceeded the distribution with a threshold of *P* < 0.05.

## Results

### Demographic, sleep, and other clinical variables

Demographic, sleep‐related, and other clinical variables are described in Table 2. No significant differences in age (*P* = 0.7), gender (*P* = 0.52), handedness (*P* = 0.61), or ethnicity (*P* = 0.34) appeared between OSA and control subjects. However, BMI values appeared significantly higher in OSA compared to control subjects (*P* = 1 × 10^−11^). PSQI, ESS, BDI‐II, and BAI also showed significant differences between groups (PSQI, *P* = 2 × 10^−17^; ESS, *P* = 2 × 10^−10^; BDI‐II, *P* = 2 × 10^−5^; BAI, *P* = 1 × 10^−5^).

**Table 2:** Demographic, neuropsychological, and sleep variables of obstructive sleep apnea (OSA) and control subjects. Age, BMI, ESS, PSQI, BDI‐II, and BAI between two groups were compared using two‐sample *t*‐tests and sex ratio between two groups was compared using the Chi‐square test

Variables	OSA (*n* = 69)	Controls (*n* = 82)	*P*‐value
Age range (years)	31–70	29–66	–
Age (mean ± SD, years)	48.3 ± 9.2	47.6 ± 9.1	0.7
Sex (Male:Female)	52:17	58:24	0.52
BMI(mean ± SD, kg/m^2^)	31.0 ± 6.2	25.1 ± 3.5	1 × 10^−11^
Handedness	9 Left; 53 Right; 7 Ambidextrous	13 Left; 64 Right; 5 Ambidextrous	0.61
Ethnicity	12 Asian; 35 White; 11 Hispanic; 9 African‐American; 1 White‐Asian; 1 Iranian‐White	23 Asian; 41 White; 10 Hispanic; 4 African‐American; 1 Asian‐White; 1 Iranian‐White; 1 Hispanic‐White; 1 El Salvador‐Hispanic	0.34
AHI (mean ± SD, events/h)	35.6 ± 23.3	−	−
ESS (mean ± SD)	9.8 ± 4.9	5.1 ± 3.5	2 × 10^−10^
PSQI (mean ± SD)	8.8 ± 4.1	3.6 ± 2.4	2 × 10^−17^
BDI‐II (mean ± SD)	8.4 ± 8.1	3.7 ± 4.9	2 × 10^−5^
BAI (mean ± SD)	9.4 ± 11.0	3.4 ± 4.5	1 × 10^−5^

AHI, apnea‐hypopnea index; BMI, body mass index; ESS, Epworth Sleepiness Scale; PSQI, Pittsburg Sleep Quality Index; BDI‐II, Beck Depression Inventory II; BAI, Beck Anxiety Inventory; SD, standard deviation.

### Whole‐brain FC

**Figure 1:**
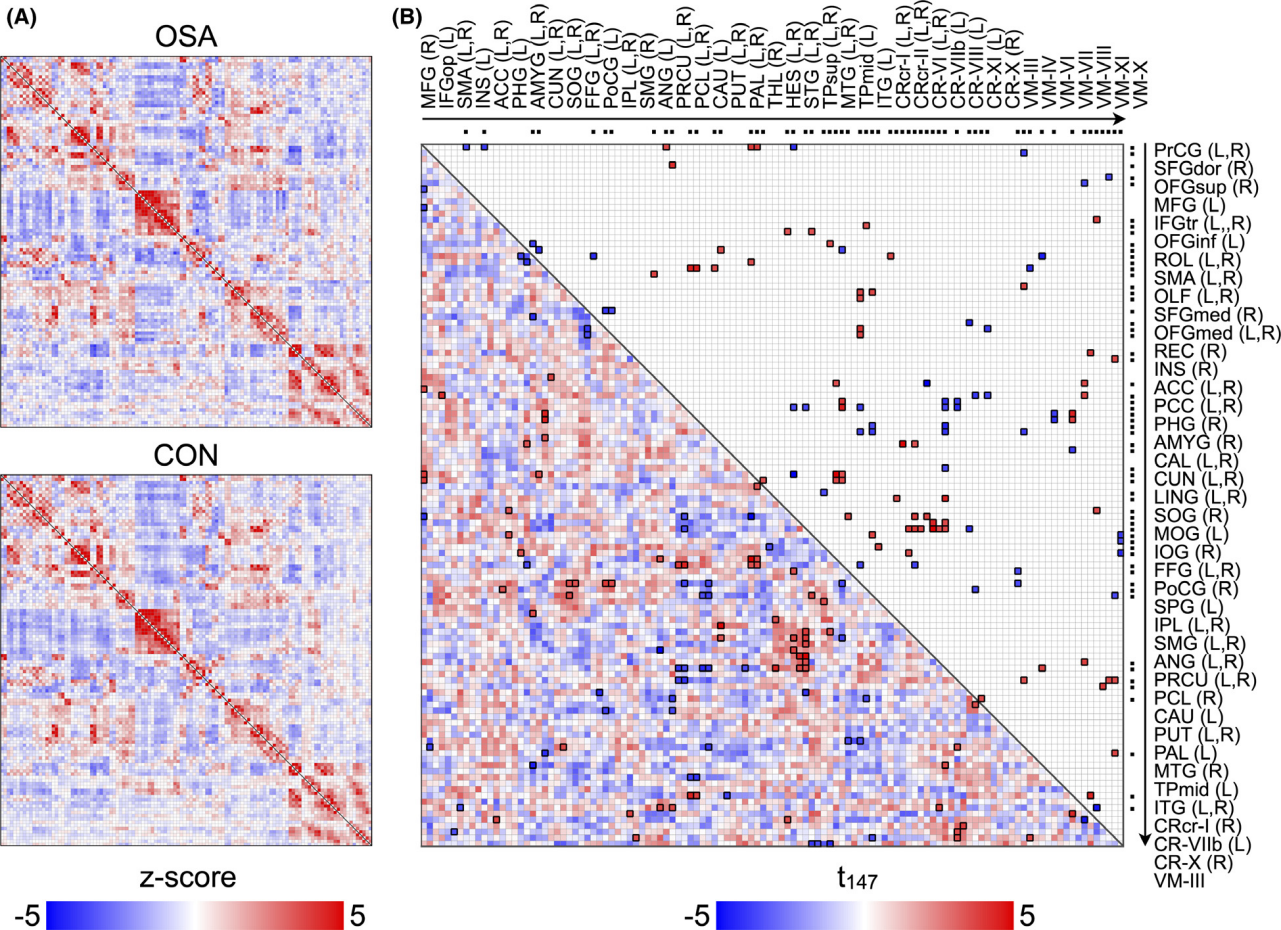
Functional connectivity matrices for each group and group comparison. (A) group‐averaging functional connectivity matrices for obstructive sleep apnea (OSA) and controls. Color bar indicates *z*‐transformed correlation coefficient and red or blue color represents positive or negative functional connectivity, respectively. (B) group‐comparing functional connectivity matrix with upper diagonal elements represents the *t*‐statistics corresponding to significantly changed functional connectivity among all pairs described in lower diagonal elements (FDR < 0.05). Red or blue color represents significantly increased or decreased functional connectivity in OSA, respectively. Dots in top and right outsides of matrix imply the regions showing significantly changed functional connectivity with one or more regions, and the regional labels are sorted by the same order of the dots. L and R indicate left and right regions. Regional brain abbreviations are listed in Table 1.

**Figure 2:**
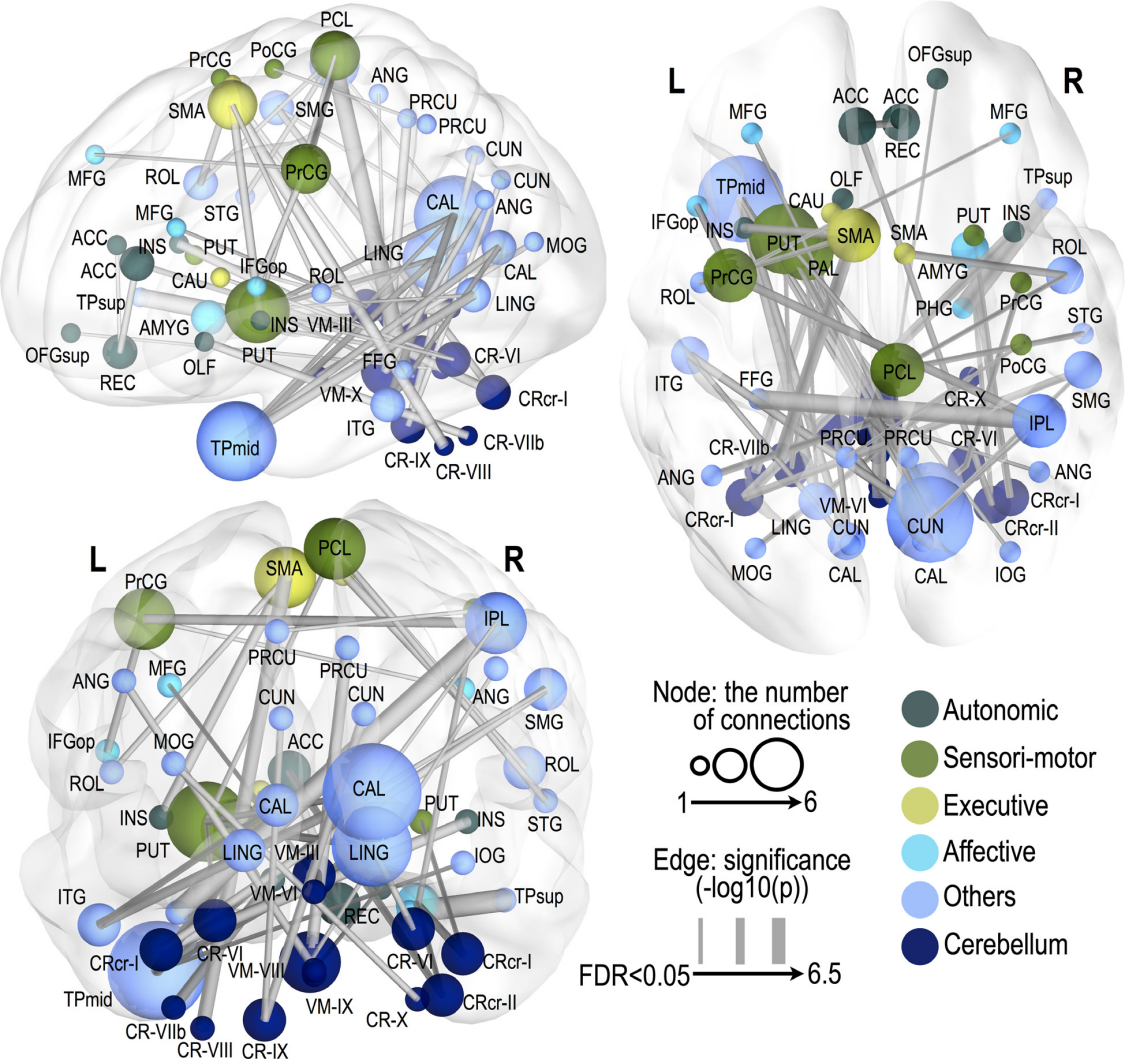
Significantly decreased functional connectivity in obstructive sleep apnea subjects. Thicker edge lines represent more significant differences, with a scale of −log10 (*P*‐value) from a minimum value to 6, surviving a threshold of FDR < 0.05. A larger nodal sphere size represents a larger number of significant edges (degree). Nodes were differentiated by different colors according to their functional categories. Regional abbreviations are listed in Table 1.

**Figure 3:**
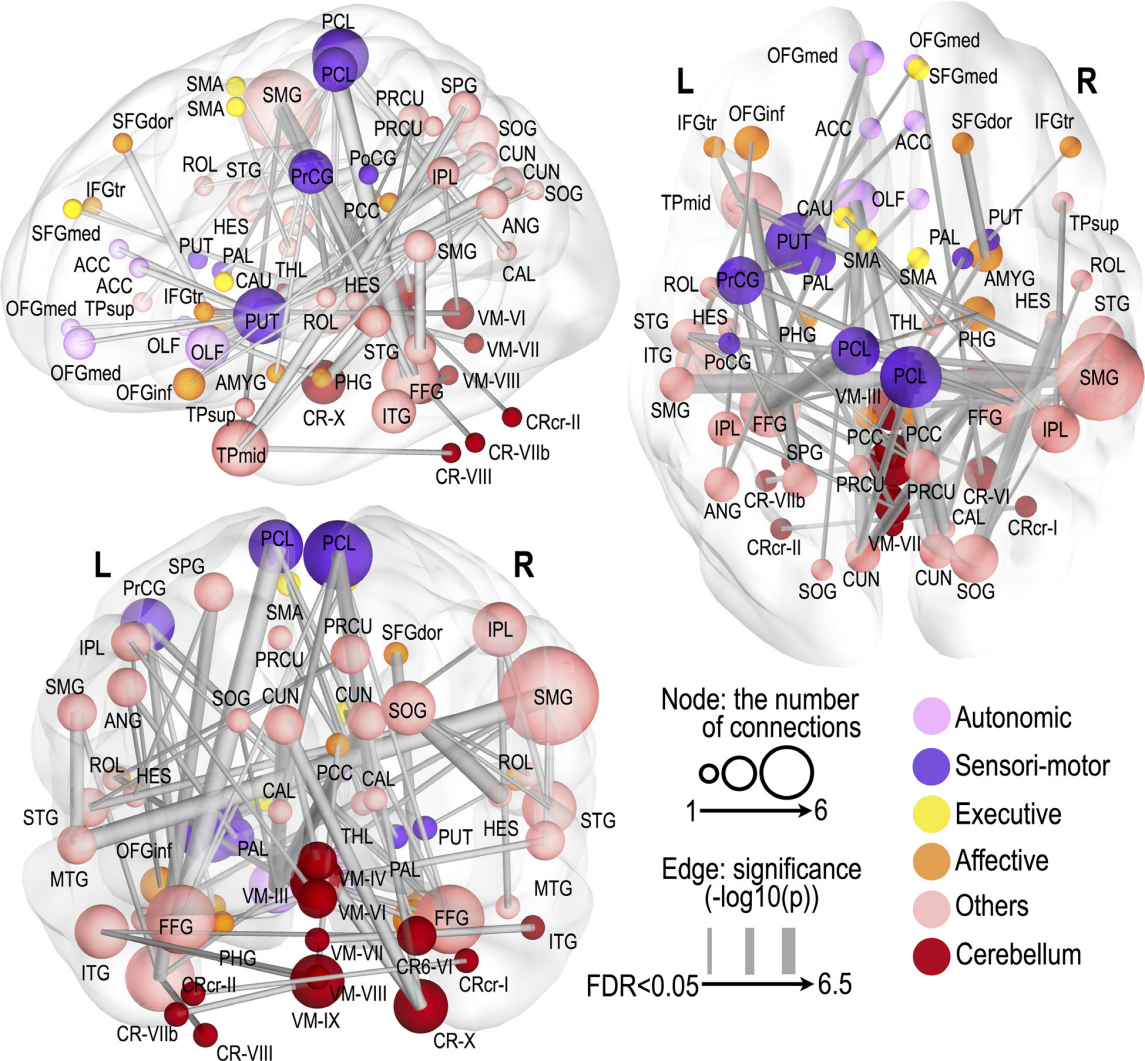
Significantly increased functional connectivity in obstructive sleep apnea subjects. Thicker edge lines represent more significant differences, with the scale of −log10 (*P*‐value) from a minimum value to 6, surviving a threshold of FDR < 0.05. Larger nodal sphere size represents a larger number of significant edges (degree). Nodes were differentiated by different colors according to their functional categories. Regional abbreviations are listed in Table 1.

Significantly altered functional connections appeared across the whole‐brain areas (FDR; *P* < 0.05) in OSA subjects. We found 49 significantly decreased and 64 significantly increased functional connections in OSA, compared to healthy control subjects. The most affected functional connections were related to cerebellar regions, but the declines were not site specific, and appeared across whole‐brain regions. Detailed FC differences between OSA and control groups are shown in figures (Figs. 1, 2, 3) and in the Supplementary materials (Tables S1 and S2).

#### Altered cerebral FC in OSA

Brain networks with reduced connectivity in OSA included interactions between the right rectus and bilateral anterior cingulate cortex (ACC), between the left insula and left supplementary motor area (SMA), between the right rectus and bilateral ACC, and between right para‐hippocampal gyrus and right superior temporal pole. Also, the left precentral gyrus in OSA showed decreased FC with the left inferior frontal gyrus, right middle frontal gyrus, and right inferior parietal lobule. The right postcentral gyrus showed reduced FC with the left precuneus, and the right paracentral gyrus with the right superior temporal gyrus. As for the motor‐related basal ganglia region, the left putamen showed reduced FC with the right paracentral lobule, right lingual, and right calcarine gyrus, and left pallidum showed reduced FC with the bilateral lingual gyri in OSA.

In contrast, enhanced FC in OSA appeared between left para‐hippocampal gyrus and right olfactory, between the right amygdala and right superior frontal gyrus, between the left amygdala and left precentral gyrus, between the right para‐hippocampal gyrus and left paracentral lobule, between the left caudate and right inferior parietal lobule, and between the left SMA and right thalamus. Also, the left putamen in OSA showed increased FC with the bilateral ACC and bilateral medial orbitofrontal gyrus (OFG), and increased connectivity also emerged between left pallidum and left medial OFG.

In addition, increased FC in OSA emerged in temporal–parietal networks (the bilateral middle/superior temporal and supramarginal gyrus, superior and middle parts of the left temporal pole, right precuneus and inferior parietal lobule, and left Heschl and superior parietal gyrus). Reduced FC (middle parts of the left temporal pole, left inferior temporal gyrus, bilateral calcarine and lingual, and right inferior occipital gyrus) or increased FC (the right superior occipital gyrus, right Rolandic, and right Heschl gyrus) also appeared in temporal–occipital networks in OSA. Lesser FC (the right inferior parietal lobule, right supramarginal gyrus, right calcarine, and left fusiform gyrus) and higher FC (the bilateral calcimine, bilateral fusiform gyrus, bilateral paracentral lobule, and left pre‐ and post‐central gyrus) were also found in parietal–occipital areas of OSA subjects.

#### Altered cerebro‐cerebellar FC in OSA

Multiple cerebellar regions showed reduced FC with cerebral brain areas, which included connections between the left cerebellar lobule VIIb and left olfactory, the right cerebellar crus II and left ACC, the left cerebellar crus I and right insula and right supramarginal gyrus, the vermis VIII and right superior OFG, the left cerebellar lobule VI and left caudate and putamen, the vermis III and left middle frontal gyrus, the right cerebellar crus I and right amygdala and putamen, the left cerebellar lobule VIII and left SMA, the right cerebellar lobule VI and right precentral as well as the lingual gyri, between the vermis IX and left pallidum, the right cerebellar lobule X and left middle occipital gyrus, the left cerebellar lobule IX and bilateral cuneus, and the vermis X and bilateral angular gyrus and right precuneus.

Several cerebellar regions also showed increased FC with cerebral brain areas, which included between the vermis III and right para‐hippocampal gyrus, amygdala, and middle temporal gyrus, between the vermis IV and left posterior cingulate cortex (PCC), between the vermis VI and left inferior frontal gyrus and inferior parietal lobule, between the vermis VII and right inferior temporal gyrus, between the vermis VIII and left inferior temporal gyrus, between the vermis IX and right PCC and left inferior temporal gyrus, between the right cerebellar lobule VI and right medial superior frontal and left inferior temporal gyri, between the left cerebellar lobule VIII and left middle temporal pole, and between the right cerebellar lobule X and bilateral cuneus.

#### Altered cerebellar FC in OSA

Within cerebellar sites, FC between the vermis III and vermis VI was significantly reduced. Increased FC was also distributed within cerebellar sites, which included connectivity between the left cerebellar crus II and right cerebellar crus I, the vermis IV and right cerebellar lobule X, as well as the vermis IX and left cerebellar lobule VIIb.

### Graph‐theoretical measures

**Figure 4:**
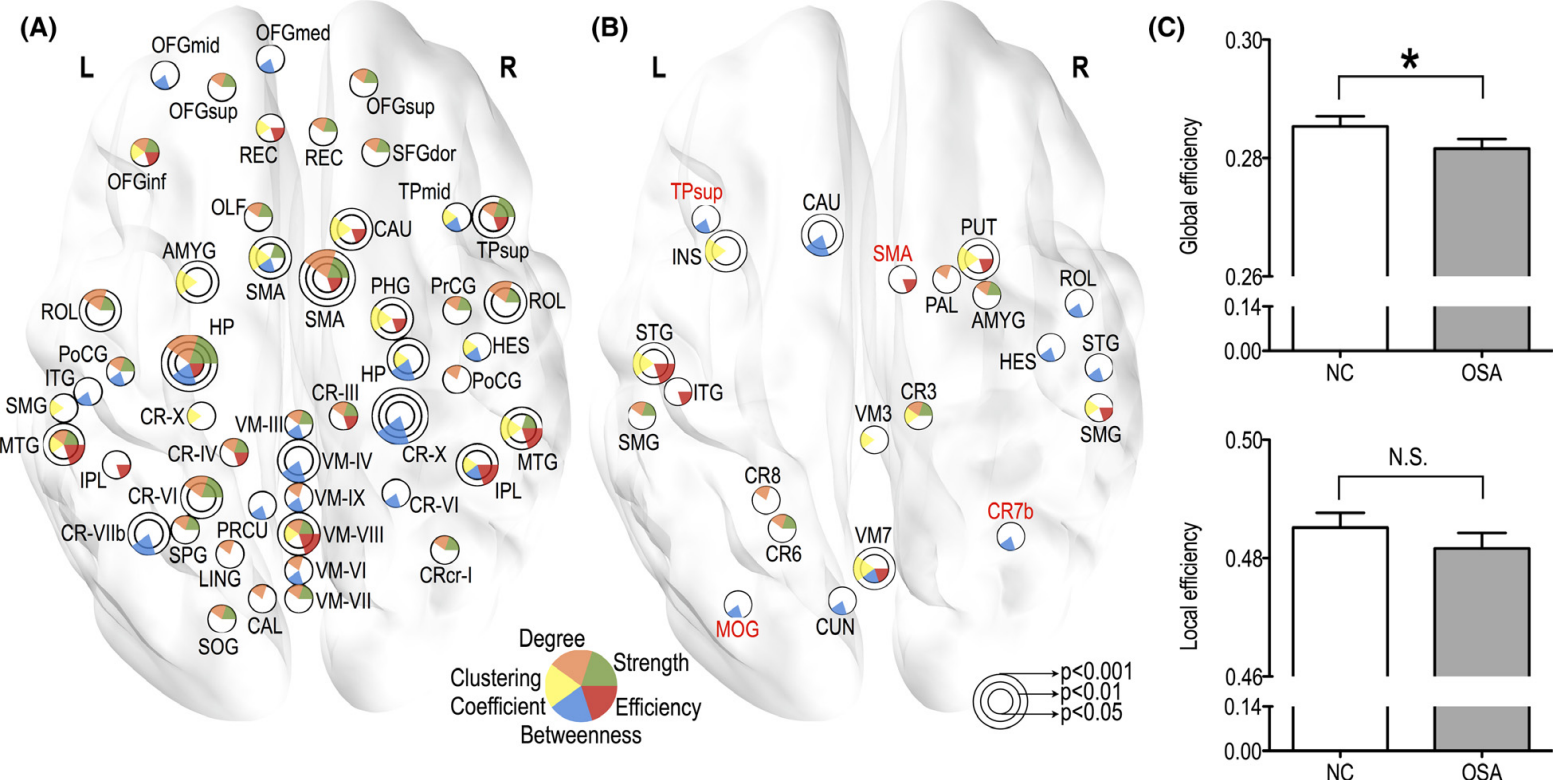
Significantly decreased global efficiency (*P* < 0.05, 10,000 permutations) and regional graph‐theoretical measures showing decreased trends in patients with obstructive sleep apnea (OSA) (*P* < 0.05 to *P* < 0.001, uncorrected, 10,000 permutations). (A) differences in nodal properties, where each color represents strength, degree, weighted clustering coefficient, betweenness, and nodal efficiency, respectively. (B) regions showing significant correlations between nodal properties and AHI values. Red colored regional labels represent negative correlations and black colored regional labels represent positive correlations. (C) differences in global properties. OSA and NC represent OSA and normal control group, respectively. Star (*) and N.S. represent significant and nonsignificant differences, respectively. Regional abbreviations are listed in Table 1.

While regional FC values showed both increased and decreased functional connections in OSA over control subjects; graph‐theoretical measures appeared with only decreased topological attributes in OSA subjects (Fig. 4). For global network properties, global efficiency showed significantly decreased values in OSA subjects (*P* = 0.04; 10,000 permutations), while local efficiency did not show any significant differences between OSA and control groups (Fig. 4C). Nodal properties showed decreased trends across whole‐brain regions (*P* < 0.05, uncorrected; 10,000 permutations). Briefly, described with a more conservative threshold of *P* < 0.01 (uncorrected; 10,000 permutations), network centrality measures declined in the bilateral hippocampus, right SMA, bilateral Rolandic operculum, and cerebellar regions (i.e., the bilateral lobule VI left lobule VIIb, right lobule X, and vermis IV). Weighted clustering coefficients of OSA subjects showed decreased values in the right para‐hippocampal gyrus, left amygdala, right caudate, left SMA, and right middle temporal gyrus. Nodal efficiency in OSA subjects was reduced in the right inferior parietal lobule, bilateral middle temporal gyrus, and vermis VIII. Detailed nodal findings are described in a Figure 4A and Table 3.

**Table 3:** Significantly decreased nodal properties in patients with obstructive sleep apnea (*P* < 0.05, uncorrected, 10,000 permutations). Each value represents the *P*‐value

Lobe	Region	Hemisphere	Degree	Strength	Betweenness	Weighted clustering coefficient	Efficiency
Frontal	SFGdor	R	0.0328	0.0266	–	–	–
	OFGsup	L	0.0282	0.0328	–	–	–
		R	0.0243	0.0423	–	–	–
	OFCmid	L	–	–	0.0143	–	–
	OFGinf	L	0.0284	0.0229		0.0243	0.0265
	OFGmed	L	–	–	0.0135		
	REC	L	–	–	–	0.0208	0.0134
		R	0.0117	0.0117	–	–	–
	OLF	L	0.046	0.0335	–	–	–
Limbic	HP	L	0.0001	0.0002	0.0087	–	0.0326
		R	–	–	0.0078	0.0447	
	PHG	R	–	–	–	0.0036	0.0122
	AMYG	L	–	–	–	0.0099	
Basal ganglia	CAU	R	–	–	–	0.0049	0.0191
Sensorimotor	SMA	L	–	0.0452	0.0221	0.0084	
		R	0.0007	0.0013	–	–	0.0216
	PrCG	R	0.0267	0.0456	–	–	–
	PoCG	L	0.0191	0.0404	0.0385	–	–
		R	0.0346		–	–	–
Parietal	SPG	L	0.043	0.0348	–	–	–
	IPL	L	–	–	–		0.0496
		R	–	–	0.0148	0.0201	0.0045
	SMG	L	–	–	–	0.03	–
	PRCU	L	–	–	0.0486	–	–
Temporal	HES	R	–	–	0.013	0.0498	–
	TPsup	R	0.0209	0.0077	–	–	0.0142
	MTG	L	0.0148	0.0117	–	0.0219	0.0017
		R	–	0.037	–	0.0056	0.0032
	ROL	L	0.0032	0.0105	–	–	–
		R	0.0081	0.0166	–	–	–
	Tpmid	R	–	–	0.0344	0.0219	–
	ITG	L	–	–	0.0355	–	–
Cerebellum	CRcr‐I	R	0.0257	0.0142	–	–	–
	CR‐III	R	0.0417	0.0397	–	–	0.0447
	CR‐IV	L	0.0205	0.0324	–	–	0.0274
	CR‐VI	L	0.002	0.0067	–	–	–
		R	–	–	0.0345	–	–
	CR‐VIIb	L	–	–	0.0029	–	–
	CR‐X	L	–	–	–	0.0304	–
		R	–	–	0.0002	–	–
	VM‐III	–	0.0364	0.0322	0.0213	–	–
	VM‐IV	–	–	–	0.006	–	–
	VM‐VI	–	0.0452	–	0.0223	–	–
	VM‐VII	–	0.0467	0.0303	–	–	–
	VM‐VIII	–	0.0468	0.0361	–	0.0423	0.0059
	VM‐IX	–	0.0238	–	0.0439	–	–
Occipital	CAL	L	0.0257	–	–	–	–
	LING	L	0.0359	–	–	–	–
	SOG	L	0.0449	0.0247	–	–	–

Regional abbreviations are listed in Table 1.

Although global topological properties (e.g., global and local efficiency) did not show any significant correlations with AHI values, significant relationships appeared between nodal properties and AHI scores in OSA subjects (Fig. 4B, *P* < 0.05, uncorrected; 10,000 permutations). AHI values were positively correlated with weighted clustering coefficients of the left insula, left superior temporal gyrus, vermis III and VII, left cerebellar lobule III and VIIb, right putamen, and right supramarginal gyrus. Degree or strength values of the left cerebellar lobule III/VI/VIII, left supramarginal gyrus, right pallidum, and right amygdala and AHI scores showed positive correlations in OSA subjects. Positive correlations emerged between AHI values and efficiency values of the left superior and inferior temporal gyrus, vermis VII, right putamen, and right suparamarginal gyrus, but negative correlation with the right SMA. We also found significantly positive correlations between AHI values and betweenness of the left caudate, left cuneus, vermis VII, right Rolandic/heschl/superior temporal regions, while negative correlations appeared between those variables of the left superior temporal pole, left middle occipital gyrus, and right cerebellar lobule VIIb.

## Discussion

We investigated whole‐brain FC and their topological attributes in OSA and control subjects using rs‐fMRI procedures. Previous studies on brain resting‐state FC in OSA subjects found local FC alterations in the middle and dorsal prefrontal cortex, left precentral gyrus, and PCC, decreased local FC in the frontal, temporal, and parietal, and increased FC in the sensorimotor, thalamic, and cerebellum regions (Santarnecchi et al. 2013; Zhang et al. 2013; Peng et al. 2014). However, previous studies were limited to within seven functionally defined subnetworks only, not including the cerebellum, with independent component analysis or limited to local FC findings (Santarnecchi et al. 2013; Zhang et al. 2013; Peng et al. 2014), and thus, cannot directly provide important topological changes in OSA brain network. A key question in this current study was how the OSA condition affects the topological properties of brain network organization, as well as individual functional interactions across the whole brain. Our findings indicate that OSA subjects have abnormal resting‐state FC in various brain regions largely related to autonomic, affective, executive, sensorimotor, and cognitive regulatory functions, areas that appeared with structural injury in previous studies of OSA subjects, as well as areas that appeared abnormal resting‐state FC (Santarnecchi et al. 2013; Zhang et al. 2013; Peng et al. 2014). Altered FC in OSA further led to disrupted topological properties, especially for integrative aspects of brain network organization.

### Cerebellar FC changes

We found reduced FC within cerebellar areas (i.e., between vermis III and VI) and between multiple cerebellar and other brain regions responsible for diverse brain functions, which may contribute to various cognitive deficiencies found in the condition. Cerebellar sites, the most structurally affected areas in OSA subjects evident from several neuroimaging studies (Macey et al. 2002, 2008; Kumar et al. 2012), showed major aberrant FC in resting conditions of OSA subjects, which was consistent with altered resting regional homogeneity in OSA cerebellum (Peng et al. 2014). As observed in previous studies, the reduced FC in OSA was distributed in the brain sites responsible for autonomic (Henderson et al. 2004; Macey et al. 2006, 2008; Joo et al. 2010, 2013; Kumar et al. 2012), cognitive process and motor/visuospatial control (Mayberg et al. 2005; Cross et al. 2008; Macey et al. 2008; Kumar et al. 2009, 2012), executive (Macey et al. 2006, 2008; Joo et al. 2010; Kumar et al. 2012), sensorimotor (Joo et al. 2013; Kumar et al. 2014a), and verbal performance (Ayalon et al. 2006). Brain regions showing increased FC were also overlapped OSA structural alterations previously reported, and cerebral brain areas increased FC with the cerebellar regions was distributed in cognitive control circuitry, responsible for memory, attention, language, and auditory processing.

The aberrant FC within cerebellar regions and between cerebellar and cerebral brain sites are responsible for diverse brain functions that may result from large alterations in white matter integrity of the projections between cerebellar and other major brain structures (Macey et al. 2008). Although the causal mechanisms altering FC remains unclear, impaired FC within the cerebellum and between the cerebellar and cerebral brain areas in OSA subjects may disrupt higher order cognitive processes. Moreover, emotion substantially affects upper airway muscle activity, demonstrating an important interaction between autonomic regulation and motor coordination. Exaggerated FC in OSA subjects may contribute to synaptic plasticity, modifying the diverse functional compensation that appears in the condition.

### Altered cerebral brain FC

#### Autonomic circuitry

In this study, altered FC in OSA subjects appeared between autonomic regulatory regions, including the ACC, OFG, rectus, putamen, and pallidum. The ACC, an area that shows gray matter volume loss, cortical thinning, and altered mean diffusivity values in OSA subjects (Macey et al. 2008; Joo et al. 2010, 2013; Kumar et al. 2012), plays an important role in autonomic and breathing regulation (Critchley et al. 2003). The medial OFG also showed structural damage (Macey et al. 2008) and altered connectivity emerged among the ACC, the rectus, and putamen regions. Connections affected in the condition also included the putamen and pallidum, showing autonomic motor deficits during blood pressure and respiratory challenges (Henderson et al. 2004; Macey et al. 2006; Kumar et al. 2012, 2014a). Thus, aberrant FC in OSA subjects may contribute to deficient autonomic regulation in the condition (Somers et al. 1995; Henderson et al. 2002; Harper et al. 2003).

#### Affective circuitry

We found altered FC between various brain areas including the insular and cingulate cortices, frontal region, hippocampus, and amygdala, sites that are involved in affective symptoms, such as depression and anxiety in OSA (Mayberg et al. 2005; Cross et al. 2008; Macey et al. 2008; Kumar et al. 2009, 2012). The ACC, an area that shows improvement in depressive signs on stimulation (Mayberg et al. 2005), showed significantly increased injury in OSA subjects with high depressive symptoms over those without such signs (Cross et al. 2008; Kumar et al. 2009). The hippocampus is structurally impaired in both adult OSA subjects (Macey et al. 2002; Morrell et al. 2003; Kumar et al. 2012) and child OSA subjects (Halbower et al. 2006), and shows more enhanced damage in OSA subjects with depressive symptoms (Frodl et al. 2002; Neumeister et al. 2005). Therefore, alterations in FC to these regions may be related to abnormal affective conditions in OSA.

#### Executive and sensorimotor circuitry

Obstructive sleep apnea subjects revealed abnormal FC in executive control regions including the ACC, caudate, and SMA. Deficient executive function is another common characteristic of OSA subjects (Bedard et al. 1991; Naegele et al. 1995). Although a primary role of the ACC is autonomic and affective control, the region also influences executive functions, and deficiencies in this area may contribute to impaired executive function in OSA subjects. Other brain areas in OSA subjects can also contribute to executive function deficits originating from neural damage in regions including the prefrontal cortex, caudate nuclei, and SMA (Macey et al. 2006, 2008; Joo et al. 2010; Kumar et al. 2012), whose configurations are well known as intrinsic executive networks, demonstrated by the resting‐state FC studies (Seeley et al. 2007).

Functional connectivity between sensorimotor regions was also differed in OSA subjects. Impairment and disease progression in these areas may reflect altered sensory input from the upper airway, including the loss of tone in the tongue musculature during inspiratory efforts of OSA. Bilateral pre‐ and post‐central gyri, areas that are involved in sensorimotor control, show cortical thinning in OSA subjects (Joo et al. 2013) and decreased resting‐state FC (Zhang et al. 2013). Other regions, including the putamen and pallidum, which influence autonomic motor control function (Saper 1982), may contribute to deficient functioning in the condition. These sites show structural injury and functional deficits during autonomic and respiratory challenges in OSA subjects (Henderson et al. 2004; Macey et al. 2006; Kumar et al. 2012, 2014a). Altered functional networks appeared in sensorimotor processing areas and may contribute to these functional impairments in the condition. However, the FC alterations during the resting state cannot be generalized outcomes during an attention task.

#### Other circuits

Altered FC among temporal, parietal, and occipital sites also appeared in OSA. Such findings are comparable with previous studies showing affected structures in OSA subjects (Macey et al. 2002, 2008; Joo et al. 2007; Yaouhi et al. 2009; Kumar et al. 2012; Zhang et al. 2013). Also, reduced brain metabolites, indicating tissue injury, in temporal, parietal, and occipital regions in OSA subjects are reported (Yaouhi et al. 2009). Among these altered functional connectivities, abnormal networks from the posterior parietal cortex, a site involved in attention processing (Chan et al. 2008; Cohen et al. 2008), may be of concern with dysfunction in the attention domain for OSA subjects. We believe that altered FC with the right inferior parietal lobule, implicated in attentional processing (Cohen et al. 2008), may account for deficient attention in OSA subjects.

### Alterations in topological attributes

The human brain is an integrative complex system, composed of functional interactions across brain regions, and brain functions are represented by optimal balance between local specialization and global integration among brain regional activities (Bullmore and Sporns 2009, 2012). Examining the overall brain network organization in disease groups can provide new insight in understanding disease pathology (Bassett and Bullmore 2009). Reduced regional metabolism by brain tissue and synaptic injury in diseased groups may lead to disrupted anatomical projection, change in FC, and eventually, an abnormal functional brain network pattern which is less effective and has reduced regional centrality in important brain regions (with compensatory increase in other regional centrality), for example, as Alzheimer's disease (Liu et al. 2014; Wang et al. 2015), Parkinson's disease (Olde Dubbelink et al. 2014), and Stroke (Yin et al. 2014). Graph‐theoretical analyses thus provided a research framework to examine brain network organization with topological properties (Bullmore and Sporns 2009, 2012).

Global integration of information (global efficiency) across whole‐brain regions was significantly reduced in OSA subjects, and a trend of less efficient regional integration was apparent, as shown in results of nodal efficiency with uncorrected significance level, but with a sufficiently repeated permutation test. Regional reductions were similarly located in regions showing abnormal FC. Specifically, regional centralities (i.e., the degree, strength, and betweenness) in OSA subjects were reduced in autonomic (orbitofrontal region), affective (hippocampus), executive (SMA), sensorimotor (pre‐ and post‐central gyri), and attention‐related areas (dorsal superior frontal gyrus and posterior parietal regions), as well as several cerebellar areas (of 26, 14 cerebellar regions), along with temporal and occipital areas. Among these areas, the left hippocampus, right SMA, left Rolandic operculum, left lobule VI, lobule VIIb, and right lobule X were strongly affected in OSA subjects. The weighted clustering coefficients, a measure of regional segregation, in OSA subjects were also reduced across the whole brain, and reductions were strongly localized in affective and executive regulatory areas (para‐hippocampal gyrus and amygdala). The regional topological values of the autonomic (i.e., the left insula and cerebellar regions), affective (i.e., the right amygdala), and executive and sensorimotor (left caudate, right SMA, right putamen, right pallidum), as well as bilateral supramarginal/temporal/left occipital regions were significantly correlated with disease severity measured as AHI variables of OSA subjects.

Reduced regional centrality of brain networks in OSA subjects emphasizes a diminished integrative and communicative hub role in the functional brain network. Reduced regional‐weighted clustering coefficients of brain networks in OSA subjects indicate weakened functional specialization as changed to local structure sparsely connected among the neighboring areas. The changes in regional topological properties of brain networks in OSA subjects, even if they show weak reduction, may entirely lead to reduced, or less efficient global integration of information. These deficiencies may be attributable to breakdown of optimal or efficient balance between functional integration and specialization by aberrant FC in the condition. However, the lack of appearance of differences in local efficiency in brain networks in OSA subjects might be due to plastic reorganization.

### Limitations and methodological considerations

Several methodological issues need to be addressed. We used nonparametric permutation tests to assess graph‐theoretical measures, while we examined whole‐brain FC in OSA over control subjects with a threshold of *P* < 0.05, FDR correction for multiple comparisons in a parametric manner. Graph‐theoretical measures did not show significant differences between OSA and control subjects, based on an FDR < 0.05 using parametric techniques. However, significantly decreased global efficiency per se implies a significant large‐scale decline in functional network organization of OSA, which may be originated from decreased nodal graph‐theoretical measures, even with weak significance level. Therefore, we documented trends of nodal properties showing a weak significance level.

We performed weight‐conserving graph‐theoretical analyses, with a network‐forming threshold of FDR < 0.05, to avoid arbitrary threshold selection needed for network binarization. Although we constructed individual brain networks with a spatial scale by parcellating the whole brain into 116 regions, widely used in brain network studies, further studies are required to compare the current findings using different spatial scales or parcellations, since their uses could reveal different graph‐theoretical results as shown in other studies (Wang et al. 2009; Fornito et al. 2010; Hayasaka and Laurienti 2010; Zalesky et al. 2010). In this study, we used temporal scale of 120 sec as relatively short length of time series, which could not fully address temporal stability, assumed in FC studies. However, network data constructed in this study may be acceptable and supplemented with a large number of subjects. We removed, through regression, the effect of global BOLD signals in calculating resting‐state FC. This procedure can effectively reduce nonneural noise (Power et al. 2014) and can improve the specificity in FC (Fox et al. 2005; Smith et al. 2009). The removal process remains a controversial issue, since those signals could potentially induce the negative correlations (Murphy et al. 2009; Chai et al. 2012). Thus, we performed additional analysis without global signal regression on FC study (Figure S1). In the additional results, overall FC patterns by group comparison were highly similar between with and without global signal regression. Also, we found highly similar increased FC patterns in OSA subjects between with and without global signal regression, while we found different decreased FC pattern – but including similar regions – in OSA. However, we regarded global signal regression as an essential noise reduction step, since global signal accounts for widely shared variance and its large fraction may be concerned with residual effects of head motion or respiration (Birn et al. 2006; Power et al. 2012; Thomas et al. 2014). Another issue that should be acknowledged is that pathological mechanisms on the increased FC outcomes in OSA is still unclear, and the authors did not verify the absence of sleep and the altered outcomes; this study was designed for single time measurement in each subject, which may not reflect on sleep‐related dysfunction. Another limitation includes nonavailability of overnight PSG data from all control subjects to diagnose any potential OSA condition. Only limited number of controls underwent for overnight PSG study, and some controls may have been included here with undiagnosed OSA condition.

## Conclusions

Recently diagnosed, treatment‐naïve OSA subjects showed complex, aberrant functional connectivities in the resting state in various brain regions regulating autonomic, affective, executive, sensorimotor, and cognitive functions. OSA‐related functional connection changes further led to disrupted topological properties, both for functional integration and specialization aspects of brain network organization. The altered FC and reorganization of brain networks may affect both parasympathetic and sympathetic interactions, as well as sensorimotor integration, all of which are affected in OSA. The functional network‐level changes likely result from the prominent structural changes in these regions described earlier in the condition.

## Conflict of Interest

None declared.

## Supporting information


**Figure S1.** Group comparison results without global signal regression.
**Table S1.** Significantly decreased functional connectivity in patients with OSA (FDR < 0.05).
**Table S2.** Significantly increased functional connectivity in patients with OSA (FDR < 0.05).
