# Supplementary material for: Disrupted functional brain network organization in patients with obstructive sleep apnea
Source: Brain Behav. 2016 Feb 1;6(3):e00441. doi: 10.1002/brb3.441 (PMC4831421; doi:10.1002/brb3.441)
Supplement: Supplementary file 1 — Figure S1. Group comparison results without global signal regression. Table S1. Significantly decreased functional connectivity in patients with OSA (FDR < 0.05). Table S2. Significantly increased functional connectivity in patients with OSA (FDR < 0.05). [file BRB3-6-e00441-s001.docx]

**SUPPLEMENTARY MATERIALS**

**Supplementary Figure S1:** Group comparison results without global signal regression. a) correlation analysis result between overall functional connectivity patterns by group comparison. b) increased functional connectivity pattern in OSA without global signal regression. c) decreased functional connectivity pattern in OSA without global signal regression. All results were applied with the same uncorrected significance level according to FDR < 0.05 in the results with global signal regression (p=0.00048 for OSA > NC, p=0.00037 for OSA < NC).


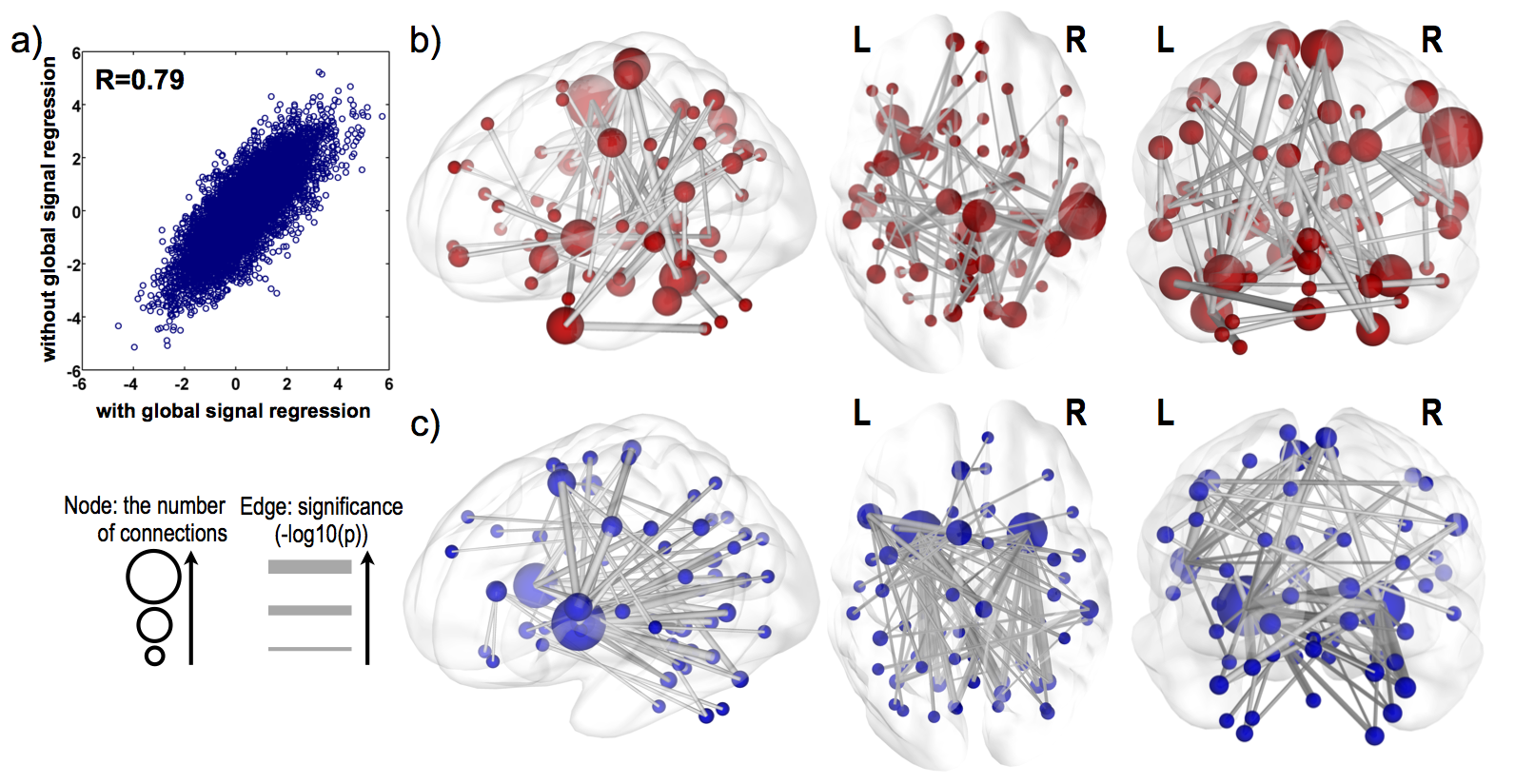


**Table S1:** Significantly decreased functional connectivity in patients with OSA (FDR < 0.05).

| **Region** | **Region** | **P-value** | **Region** | **Region** | **P-value** |
| --- | --- | --- | --- | --- | --- |
| Right MFG | Left PrCG | 3.0E-04 | Left ITG | Left CAL | 1.3E-04 |
| Left IFGop | Left PrCG | 7.4E-05 |  | Right CAL | 3.2E-05 |
| Left SMA | Left ROL | 1.4E-04 | Left CRcr-I | Right INS | 1.7E-05 |
| Right SMA | Right ROL | 4.7E-05 |  | Right SMG | 5.2E-05 |
| Left INS | Left SMA | 2.5E-05 | Right CRcr-I | Right AMYG | 6.4E-05 |
| Left ACC | Right REC | 6.1E-05 |  | Right PUT | 2.5E-04 |
| Right ACC | Right REC | 2.5E-04 | Right CRcr-II | Left ACC | 9.3E-05 |
| Right IPL | Left PrCG | 3.2E-05 |  | Right AMYG | 2.0E-04 |
|  | Right CAL | 3.6E-04 | Left CR-VI | Left CAU | 2.9E-04 |
|  | Left FFG | 4.1E-07 |  | Left PUT | 2.6E-04 |
| Right SMG | Right CAL | 2.7E-04 | Right CR-VI | Right PrCG | 1.4E-04 |
| Left PRCU | Right PoCG | 3.2E-04 |  | Right LING | 1.1E-04 |
| Right PCL | Right ROL | 2.0E-04 | Left CR-VIIb | Left OLF | 2.5E-05 |
| Left PUT | Right CAL | 1.5E-04 | Left CR-VIII | Left SMA | 6.3E-06 |
|  | Right LING | 1.9E-04 | Left CR-IX | Left CUN | 1.2E-04 |
|  | Right PCL | 2.2E-04 |  | Right CUN | 4.9E-05 |
| Left PAL | Left LING | 3.5E-04 | Right CR-X | Left MOG | 1.1E-04 |
|  | Right LING | 1.3E-04 | VM-III | Left MFG | 3.0E-04 |
| Right STG | Right PCL | 1.3E-04 | VM-VI | VM-III | 3.1E-06 |
| Right TPsup | Right PHG | 2.9E-07 | VM-VIII | Right OFGsup | 3.7E-04 |
| Left TPmid | Left CAL | 4.9E-05 | VM-IX | Left PAL | 2.4E-04 |
|  | Right CAL | 2.1E-04 | VM-X | Left ANG | 6.3E-05 |
|  | Left LING | 1.5E-05 |  | Right ANG | 3.2E-04 |
|  | Right LING | 6.4E-05 |  | Right PRCU | 1.5E-05 |
|  | Right IOG | 3.2E-04 |  |  |  |

**Table legend:** Regional abbreviations are listed in Table 1.

**Table S2:** Significantly increased functional connectivity in patients with OSA (FDR < 0.05).

| **Region** | **Region** | **P-value** | **Region** | **Region** | **P-value** |
| --- | --- | --- | --- | --- | --- |
| Left PHG | Right OLF | 1.4E-04 | Right HES | Right SOG | 1.3E-06 |
| Left AMYG | Left PrCG | 2.6E-04 | Left STG | Right SMG | 7.3E-05 |
| Right AMYG | Right SFG | 2.7E-05 |  | Right PRCU | 4.3E-04 |
| Left CUN | Left OLF | 6.8E-06 | Right STG | Right SOG | 4.6E-04 |
| Right CUN | Left OLF | 6.7E-06 |  | Right IPL | 1.2E-04 |
| Left SOG | Left OLF | 3.4E-04 |  | Right SMG | 6.7E-05 |
| Right SOG | Right ROL | 4.0E-04 | Left TPsup | Right SMG | 2.0E-04 |
| Left FFG | Left PrCG | 6.6E-05 | Right TPsup | Right IPL | 1.4E-04 |
|  | Right SMA | 3.8E-04 | Left MTG | Left SMG | 1.1E-05 |
| Right FFG | Left PrCG | 9.2E-05 |  | Right SMG | 5.6E-07 |
| Left PoCG | Right FFG | 4.5E-04 | Right MTG | Right SMG | 3.9E-04 |
| Left IPL | Left OFGinf | 4.8E-04 | Left TPmid | Left SPG | 1.1E-05 |
| Left ANG | Left OFGinf | 4.5E-04 |  | Left SMG | 2.1E-04 |
| Right PRCU | Left ROL | 4.4E-04 |  | Right SMG | 2.4E-04 |
| Left PCL | Right PHG | 3.8E-04 | Left CRcr-II | Right CRcr-I | 3.1E-04 |
|  | Left FFG | 4.2E-07 | Right CR-VI | Right SFGmed | 2.2E-04 |
|  | Right FFG | 2.2E-04 |  | Left ITG | 4.2E-04 |
| Right PCL | Left CAL | 7.1E-05 | Left CR-VIII | Left TPmid | 1.8E-04 |
|  | Right CAL | 7.8E-06 | Right CR-X | Left CUN | 9.2E-06 |
|  | Left FFG | 4.1E-04 |  | Right CUN | 1.3E-04 |
|  | Right FFG | 2.9E-04 | VM-III | Right PHG | 2.5E-04 |
| Left CAU | Right IPL | 3.4E-04 |  | Right AMYG | 2.2E-04 |
| Left PUT | Left OFGmed | 2.5E-04 |  | Right MTG | 1.2E-04 |
|  | Right OFGmed | 3.8E-04 | VM-IV | Left PCC | 1.2E-04 |
|  | Left ACC | 5.5E-05 |  | Right CR-X | 1.3E-05 |
|  | Right ACC | 1.9E-04 | VM_VI | Left IFGtr | 1.6E-04 |
| Right PUT | Right IFGtr | 4.7E-04 |  | Left IPL | 3.1E-04 |
| Left PAL | Left OFGmed | 2.2E-04 | VM-VII | Right ITG | 9.8E-05 |
|  | Left ANG | 1.6E-04 | VM-VIII | Left ITG | 2.9E-04 |
| Right PAL | Left PRCU | 3.1E-04 | VM-IX | Right PCC | 2.3E-04 |
| Right THL | Left SMA | 3.0E-04 |  | Left ITG | 2.9E-04 |
| Left HES | Left SPG | 3.9E-04 |  | Left CR-VIIb | 1.7E-04 |

**Table legend:** Regional abbreviations are listed in Table 1.
